# Pd on cyclotriphosphazen-hexa imine decorated boehmite as an efficient catalyst for hydrogenation of nitro arenes under mild reaction condition

**DOI:** 10.1038/s41598-022-19288-0

**Published:** 2022-09-03

**Authors:** Samahe Sadjadi, Neda Abedian-Dehaghani, Majid M. Heravi

**Affiliations:** 1 Gas Conversion Department, Faculty of Petrochemicals, Iran Polymer and Petrochemical Institute, PO Box 14975-112, Tehran, Iran; 2 Department of Chemistry, School of Physics and Chemistry, Alzahra University, Vanak, PO Box 1993891176, Tehran, Iran

**Keywords:** Chemistry, Catalysis, Inorganic chemistry

## Abstract

Cyclotriphosphazen-hexa imine ligand was prepared through successive reactions of phosphonitrilchloride trimer with 4-hydroxybenzaldehyde and (3-aminopropyl) triethoxy silane. The as-prepared ligand was then covalently grafted on boehmite to furnish an efficient support for the immobilization of Pd nanoparticles and synthesis of a novel heterogeneous catalyst for hydrogenation of nitro arenes. The catalytic tests revealed that the catalyst had excellent catalytic activity for hydrogenation of various nitro arenes with different steric and electronic features under mild reaction condition in aqueous media. Noteworthy, the catalyst was highly selective and in the substrates with ketone or aldehyde functionalities, reduction of nitro group was only observed. The catalyst was also recyclable and only slight loss of activity was detected after each recycling run. Hot filtration test also approved true heterogeneous nature of catalysis.

## Introduction

**Figure 1:**
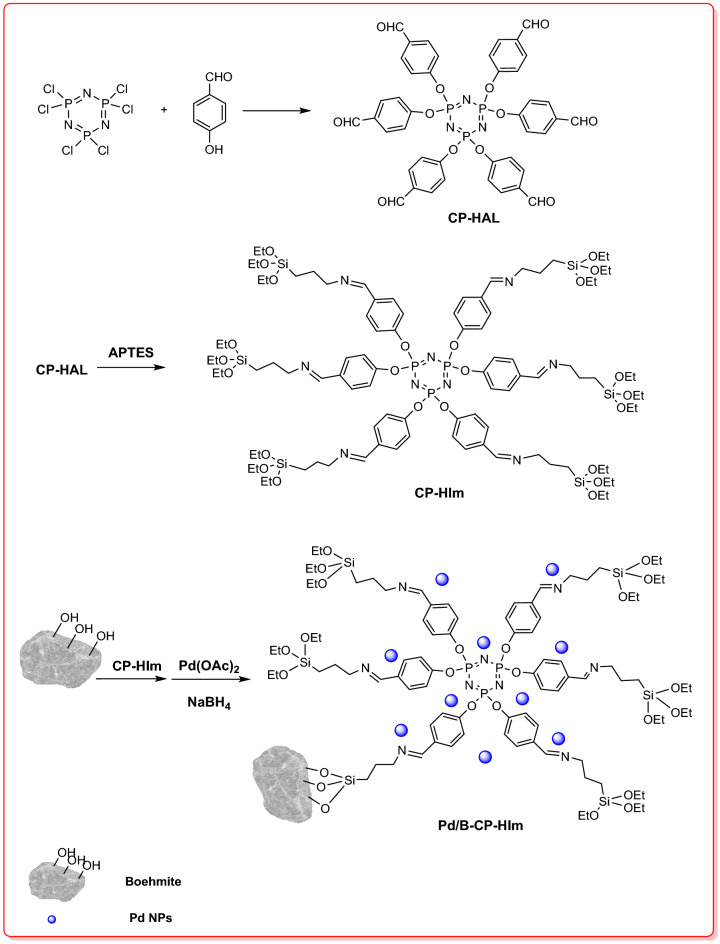
Pictorial procedure for the synthesis of Pd/B-CP-HIm.

Natural compounds are interesting candidates for industrial applications due to their availability, bio-degradability, bio-compatibility and relatively low-cost^1,2^. These compounds have also attracted considerable attention^3,4^ in catalysis and up to now, myriad catalysts for diverse range of chemical transformations have been developed using various types of natural compounds ranging from carbohydrates to clays^5,6^. Some of the reported natural-based catalysts exhibited comparable or even superior activity compared to their synthetic counterparts. Natural compounds can be utilized either as the main catalytic species or as catalyst supports^7,8^. Moreover, they can be applied as potent reducing or capping agents for the synthesis of nanoparticles (NPs)^9^. To improve the performance of natural compounds, it is necessary to modify them chemically or physically prior to use. Using these modifications, the textural properties, as well as the surface chemistry of natural compounds can be tuned. One of the natural compounds that showed promising results for the catalytic applications is boehmite (γ-AlOOH)^10^ that is a non-toxic and thermally and chemically stable compound and can be found in nature in large quantity^10,11^. Boehmite that possesses multiple –OH groups on its surface can also be chemically modified by introduction of various chemical functionalities via covalent or non-covalent approaches^12^. Hence, it is possible to adjust its properties by using proper functional groups. Functionalization of boehmite is more highlighted when immobilization of metallic nanoparticles is targeted. In this case, use of efficient organic ligands^13^, such as phosphonitrilchloride trimer that contains various heteroatoms and can efficiently immobilize NPs and provides high dispersion of metallic NPs is of great importance^14^. Specially, in catalysts, in which precious metals, such as Pd nanoparticles are utilized, introduction of functional groups on the supporting materials for effective anchoring of metallic particles and suppressing their leaching is remarkably important from economic and environmental points of view. As an example, in hydrogenation catalysts that mostly contain precious metals^15,16^, supporting materials are mostly functionalized by multi-heteroatom based ligands to suppress leaching of the immobilized precious metal. Among various types of hydrogenation reactions, hydrogenation of nitro arenes has received immense attention due to the wide range of utility of aniline derivatives^17^. Considering these issues and following our attempts to devise efficient catalytic supports using natural compounds^18–20^, herein we would like to report a new supporting compound, B-CP-HIm, prepared from chemical functionalization of boehmite by a cyclotriphosphazen-hexa imine trimer-based ligand, synthesized via reaction of phosphonitrilchloride trimer with 4-hydroxybenzaldehyde and (3-aminopropyl) triethoxy silane. The as-prepared support was then applied for the stabilization of Pd NPs, Fig. 1, and developing an efficient catalyst for the hydrogenation of nitro-compounds under mild reaction condition.

## Result and discussion

### Analysis of Pd/B-CP-HIm

**Figure 2:**
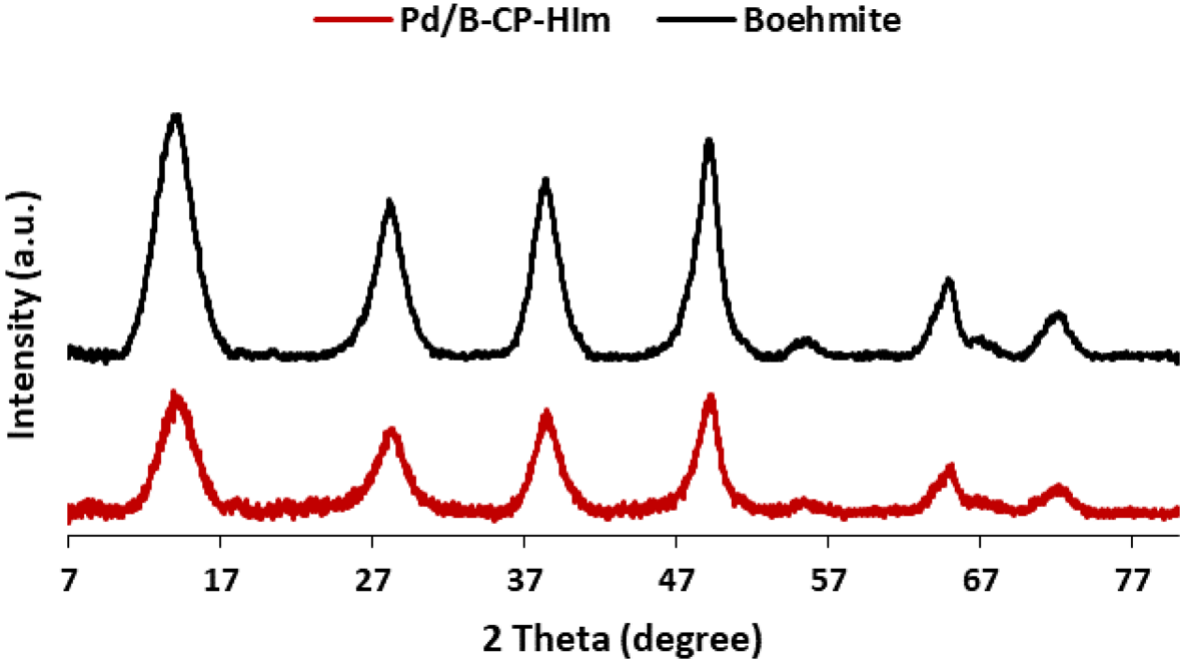
XRD patterns of boehmite and Pd/B-CP-HIm.

As discussed, CP-HIm ligand has been prepared and then conjugated on boehmite to furnish an efficient support for Pd stabilization. It was expected that boehmite maintained its structure upon grafting of CP-HIm ligand and Pd immobilization. To verify this issue, XRD patterns of boehmite and Pd/B-CP-HIm were recorded. As depicted in Fig. 2, the two XRD patterns are similar and the characteristic peaks of boehmite (2θ = 14.62°, 28.92°, 39.10°, 49.16°, 55.32°, 65.26° and 72.38°)^21^ are observable in the XRD pattern of Pd/B-CP-HIm. Worth mentioning, the peaks discerned in the XRD pattern of Pd/B-CP-HIm appeared at 2θ values exactly similar to that of boehmite, indicating the stability of boehmite in the course of chemical modification. XRD analysis and comparison of the two recorded patterns showed that Pd NPs peaks were not observable, indicating that the formed NPs were fine and well-dispersed^22^.

**Figure 3:**
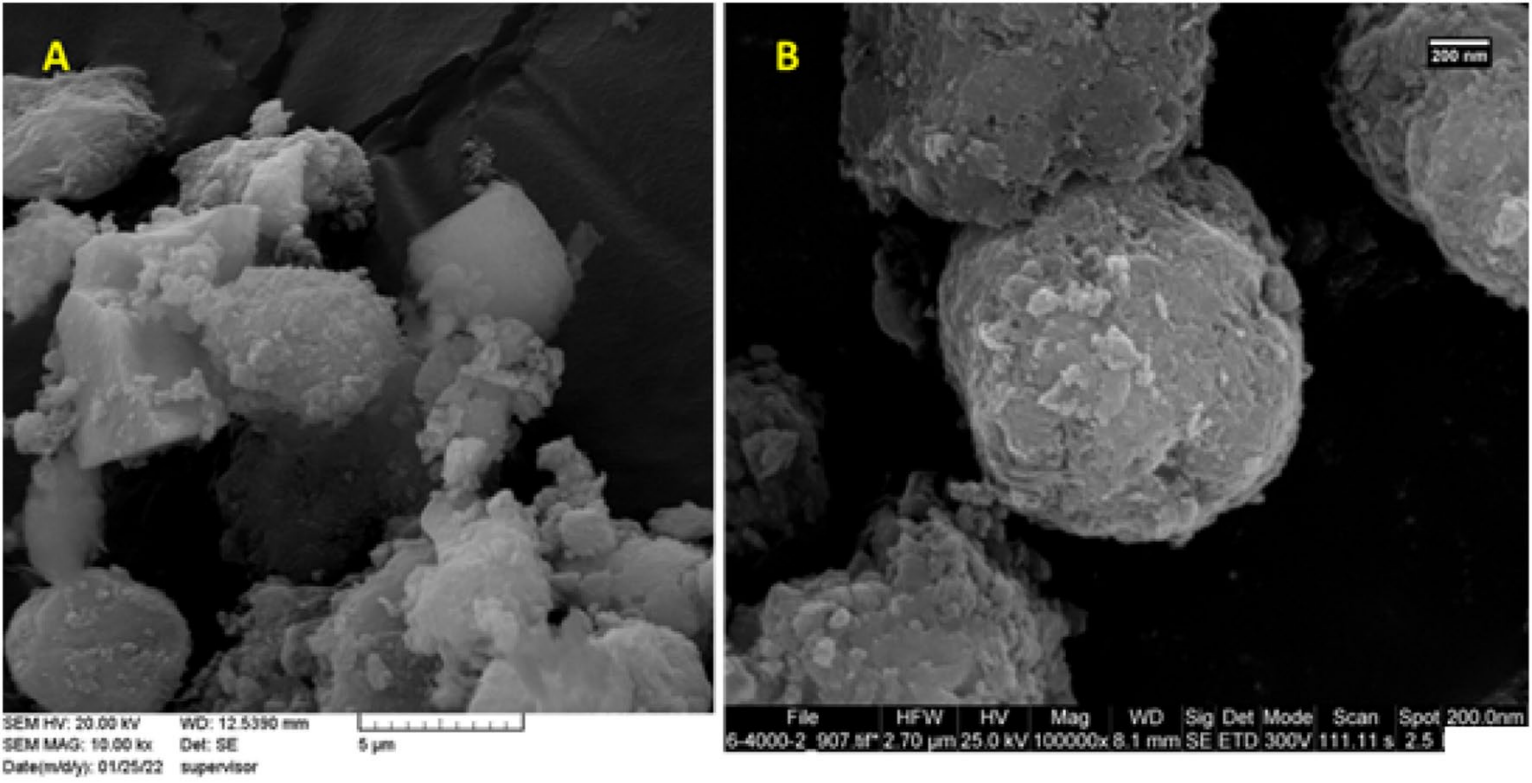
SEM images of (**A**): boehmite and (**B**): Pd/B-CP-HIm.

To study the possible morphological changes upon introduction of CP-HIm and Pd NPs, SEM images of boehmite and Pd/B-CP-HIm were compared, Fig. 3. As presented, both boehmite and Pd/B-CP-HIm showed orthorhombic cubic morphology, indicating that chemical modification did not affect the morphology of the catalyst remarkably.

**Figure 4:**
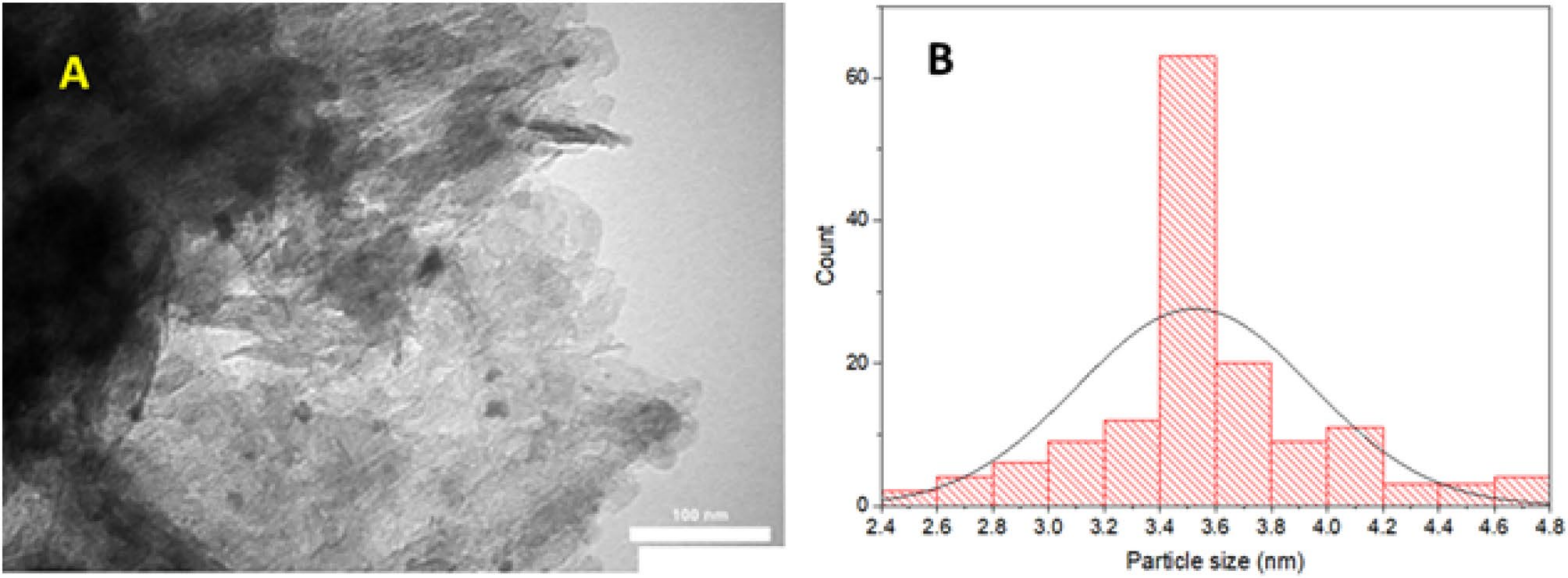
(**A**) TEM image of Pd/B-CP-HIm and (**B**) particle size distribution curve for Pd NPs.

To further shed light onto the morphology of Pd/B-CP-HIm, its TEM images were recorded. As illustrated in one of the selected TEM images, Fig. 4, Pd NPs were well-distributed on the as-synthesized B-CP-HIm support with no aggregation. Moreover, the average particle size of Pd NPs was estimated to be 3.50 ± 0.42 nm.

**Figure 5:**
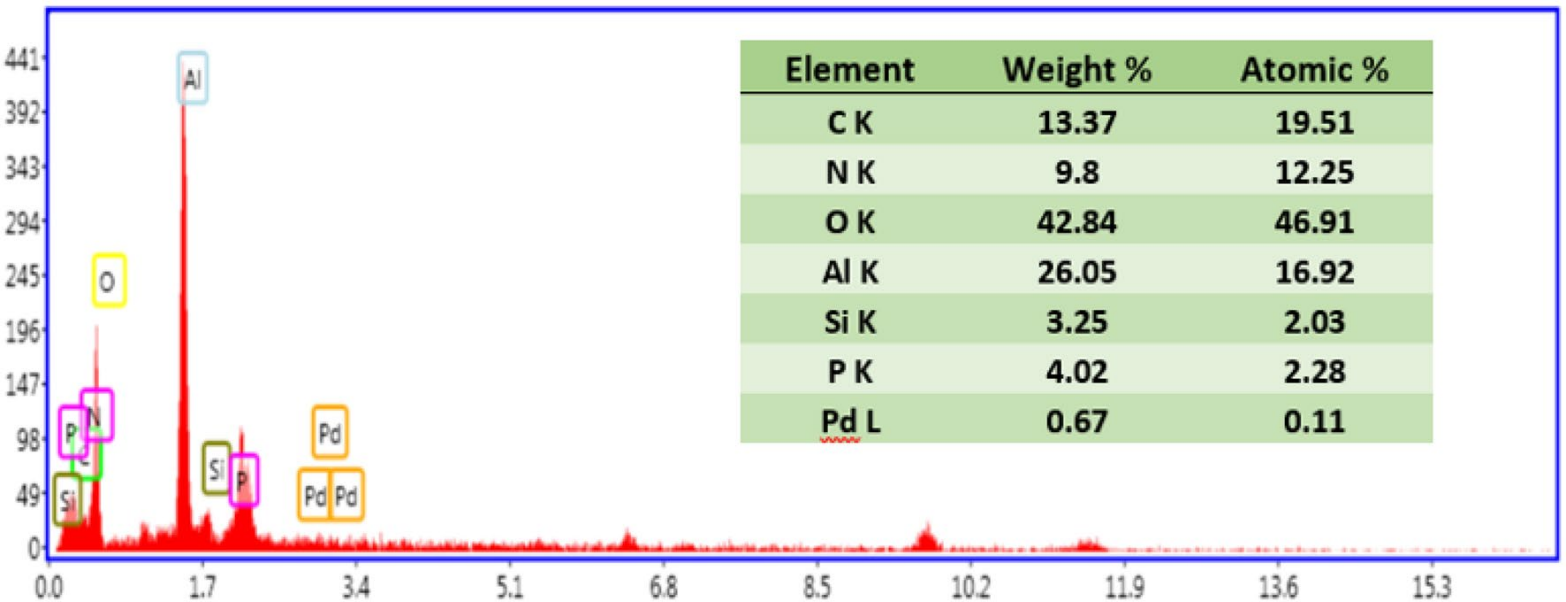
EDS analysis of Pd/B-CP-HIm.

**Figure 6:**
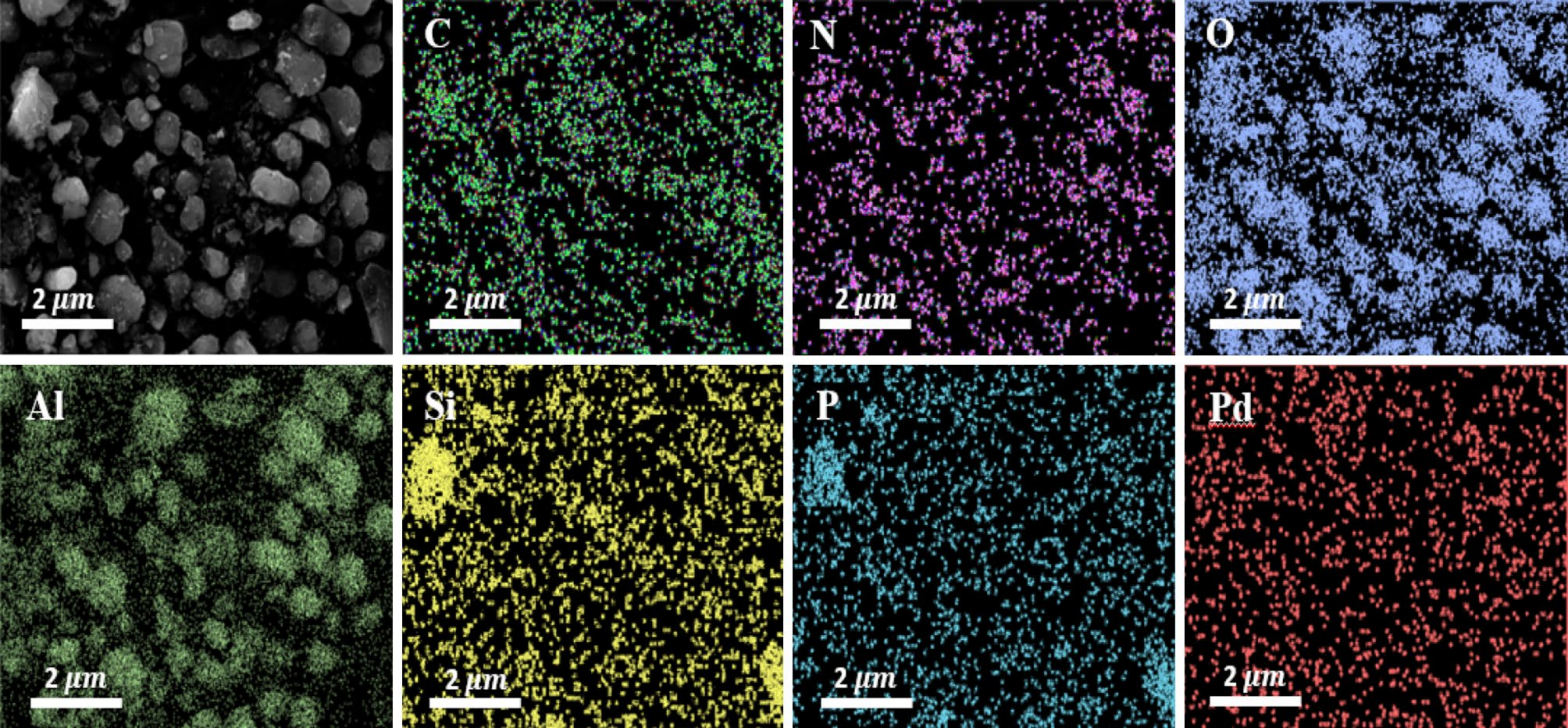
Elemental mapping analysis of Pd/B-CP-HIm.

To confirm conjugation of the as-synthesized HIm ligand and also homogeneous dispersion of Pd NPs, EDS and elemental mapping analyses were accomplished, Figs. 5 and 6. As displayed, C, N, O, Al, Si, P and Pd atoms were detected in EDS analysis, among which, Al and O are the elements present in the boehmite structure and P, N, O, C, Si atoms are representative of CP-HIm ligand, Fig. 5. Elemental mapping analysis also showed that Pd and the ligands atoms (P, N, O, C, Si atoms) are distributed almost uniformly, Fig. 6.

**Figure 7:**
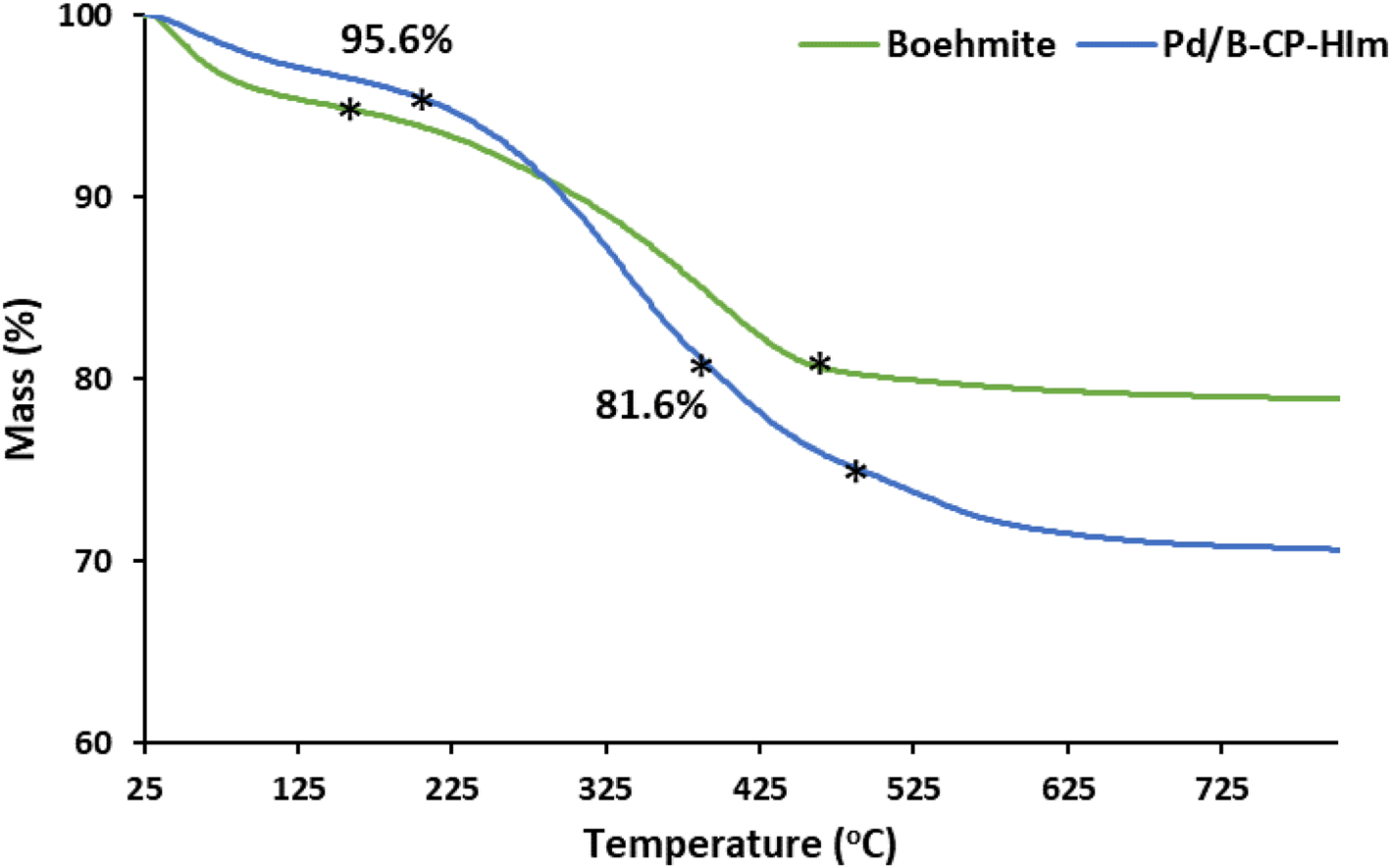
TG curves of boehmite and Pd/B-CP-HIm.

TGA not only can show the thermal stability of Pd/B-CP-HIm, but also can be used to approve grafting of CP-HIm ligand on boehmite. To this purpose, the thermograms of boehmite and Pd/B-CP-HIm were compared, Fig. 7. In boehmite thermogram, loss of structural water at low temperature (~ 150 °C) and decomposition at elevated temperature (~ 450 °C) were detected. In the Pd/B-CP-HIm thermogram, however, three weight loss steps were observed, two of them were related to the loss of water and boehmite decomposition and one of them (detected at 380 °C, 14 wt.%) was due to the decomposition of CP-HIm. In fact, this additional weight loss step approves conjugation of the organic ligand on boehmite.

**Figure 8:**
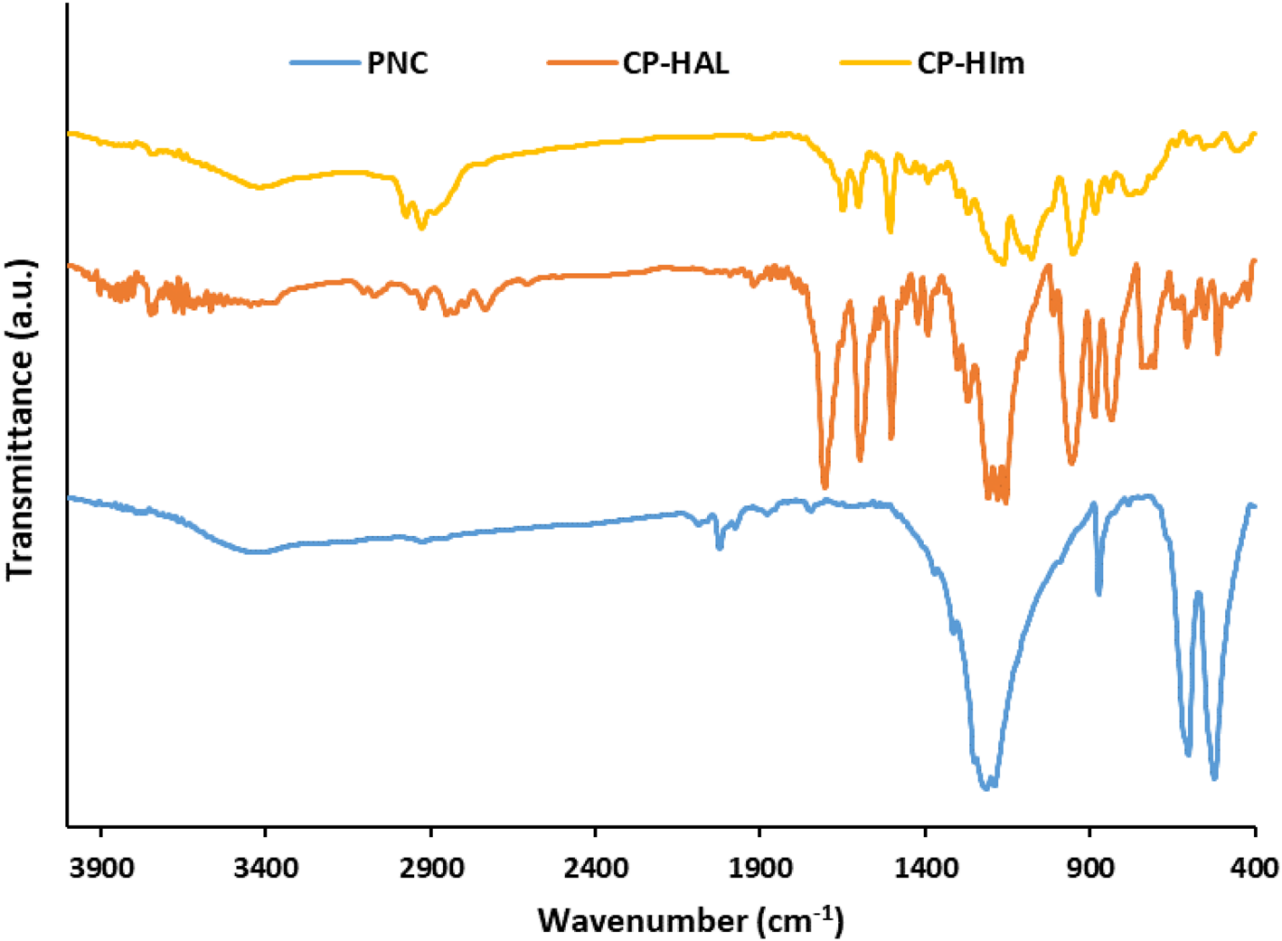
FTIR spectra of boehmite, PNC, CP-HAL, CP-HIm and Pd/B-CP-HIm.

Formation of Pd/B-CP-HIm was also verified via FTIR spectroscopy, Fig. 8. To have a better understanding, FTIR spectrum of Pd/B-CP-HIm was compared with that of boehmite, PNC, CP-HAL, CP-HIm and Pd/B-CP-HIm. As seen, FTIR spectrum of boehmite is in good accordance with the literature and showed the absorbance bands at 3080 and 3380 cm^−1^ due to symmetric and asymmetric vibrations of the O–H bonds^23,24^, 480 cm^−1^, 605 cm^−1^ and 735 cm^−1^ related to Al–O bands absorption^12^ and 1070 cm^−1^ and 1161 cm^−1^ as a result of vibrations of hydrogen bonds of –OH moieties^25^. In the case of PNC, the vibration of band in the range of 1190 cm^−1^ to 1213 cm^−1^ is ascribed to the asymmetric vibration of (P = N–P)^26,27^ and the absorbance bands at 520 cm^−1^ and 601 cm^−1^ are attributed to P–Cl bands^28^ in the framework of PNC^29^. In the FTIR spectrum of CP–HAL, the stretching vibration around 1700 cm^−1^ is attributed to –C = O bond. The peak around 1500 cm^−1^ is related to the benzene ring vibration (Ph-O) and the peak at ~ 970 cm^−1^ is related to the stretching vibration of (P-O-C) that approves replacement of the phenoxy group with chlorine atoms^28^. Also, the aromatic C–H bands were detected in 3000–3100 cm^−1^^27^. In the FTIR spectrum of CP-HIm, the peak at 1700 cm^−1^ disappeared and the bands at 1600–1650 cm^−1^ appeared, indicating the formation of imine bond^27^. Also, the aliphatic stretching vibration of C-H bands of APTES are clearly distinguishable at 2850–2974 cm^−1^^26^. In the FTIR spectrum of Pd/B-CP-HIm, all of the characteristic peaks of CP-HIm and boehmite are detectable, confirming successful linkage of CP-HIm to the boehmite support.

According to the ICP analysis, Pd content in Pd/B-CP-HIm was 1.5 wt.%.

### Catalyst activity

As Pd/B-CP-HIm contains Pd NPs in its structure, it can be considered as a versatile catalyst for promoting various chemical transformations, such as hydrogenation. As a model Pd-catalyzed reaction, hydrogenation of nitro-compounds to the corresponding aniline derivatives has been targeted. As some of the parameters, such as Pd/B-CP-HIm dosage, reaction solvent and temperature can affect the yield of the reaction, optimized values of these parameters were first found by repeating a model reaction (hydrogenation of nitro benzene) under different reaction conditions, Table 1. As presented, the first test was performed in water as solvent at 40 °C in the presence of 10 mg Pd/B-CP-HIm. The result showed that under the aforesaid condition, moderate yield of aniline was furnished. To enhance the yield of aniline, the reaction temperature was elevated from 40 to 45 °C. As shown, upon increase of the reaction temperature, the yield of aniline increased. To further elucidate the role of reaction temperature in the reaction yield, the model reaction was repeated at 50 and 55 °C. As tabulated, the reaction yield increased by elevating temperature to 50 °C. However, aniline yield remained constant upon further increase of the reaction temperature. Therefore, the optimum value for reaction temperature was considered as 50 °C. Next, the effect of reaction solvent was studied by performing the model reaction in EtOH/H_2_O (1/1), THF, CH_3_CN and CHCl_3_ as solvents. As the result revealed, performing the reaction in organic solvents led to lower yields of the desired products, while using EtOH/H_2_O (1/1) led to higher yield of aniline than water and selected as the solvent of choice. Finally, the effect of Pd/B-CP-HIm dosage was appraised by repeating the model reaction in the presence of 10–40 mg catalyst. As depicted, increase of this value from 10 to 30 mg led to the increase of the reaction yield. However, use of 40 mg Pd/B-CP-HIm did not result in any difference and the optimum value of the catalyst was 30 mg. As shown, under the optimum reaction condition, i.e. use of 30 mg Pd/B-CP-HIm, in EtOH/H_2_O (1/1) at 50 °C aniline was furnished in 100% yield after 2 h.

**Table 1:** Optimization of the reaction condition for the hydrogenation of nitrobenzene.

Entry	Pd/B-CP-HIm (mg)	Solvent	Temp. (°C)	Yield (%)
1	10	H_2_O	40	50
2	10	H_2_O	45	55
3	10	H_2_O	50	65
4	10	H_2_O	55	65
5	10	EtOH/H_2_O (1/1)	50	73
6	10	THF	50	57
7	10	CH_3_CN	50	65
8	10	CHCl_3_	50	60
9	20	EtOH/H_2_O (1/1)	50	85
10	30	EtOH/H_2_O (1/1)	50	100
11	40	EtOH/H_2_O (1/1)	50	100

As discussed above, Pd/B-CP-HIm exhibited excellent activity for the hydrogenation of nitrobenzene. To assay whether this methodology can be generalized to other nitro-compounds, a variety of nitroarene derivatives with electron donating and electron withdrawing groups were subjected to hydrogenation reaction under the optimized reaction condition. Gratifyingly, the results, Table 2, confirmed that various nitroarenes with different electronic and steric properties can be hydrogenized to give the corresponding products in excellent yields. Notably, sterically demanding derivatives and less active nitroarene derivatives, i.e. the derivatives with electron donating groups, led to slightly lower yields. It is worth mentioning that Pd/B-CP-HIm was selective towards hydrogenation of –NO_2_ functionality and in the substrates with –C = O groups (Table 2, entries 2 and 3), the carbonyl moiety remained intact and only reduction of nitro functionality was observed.

**Table 2:**
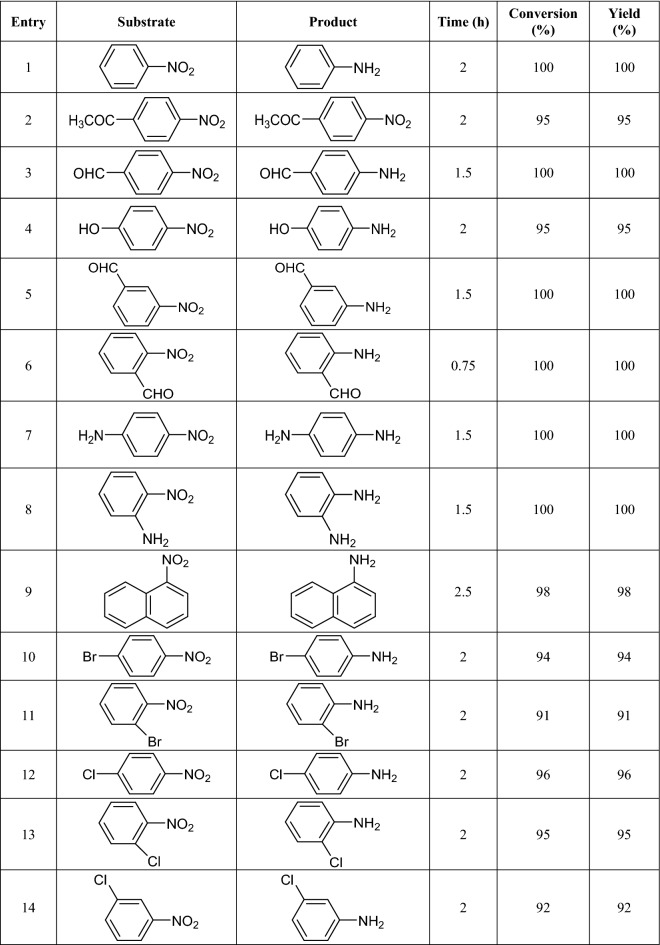
Hydrogenation reaction of various nitro aromatic substrates catalyzed by Pd/B-CP-HIm^a^.

^a^Reaction condition: nitro aromatic substrates (1 mmol), Pd/B-CP-HIm (30 mg), EtOH/H_2_O; 1/1(6 mL), 50 °C, H_2_ (1 atm).

### Recyclability

**Figure 9:**
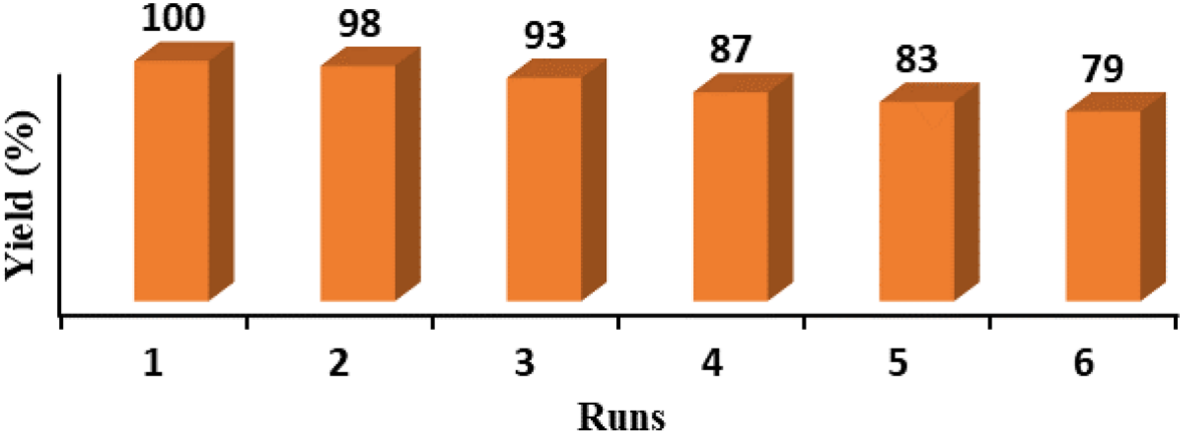
Recyclability of Pd/B-CP-HIm for the model reactions under the optimum reaction condition.

**Figure 10:**
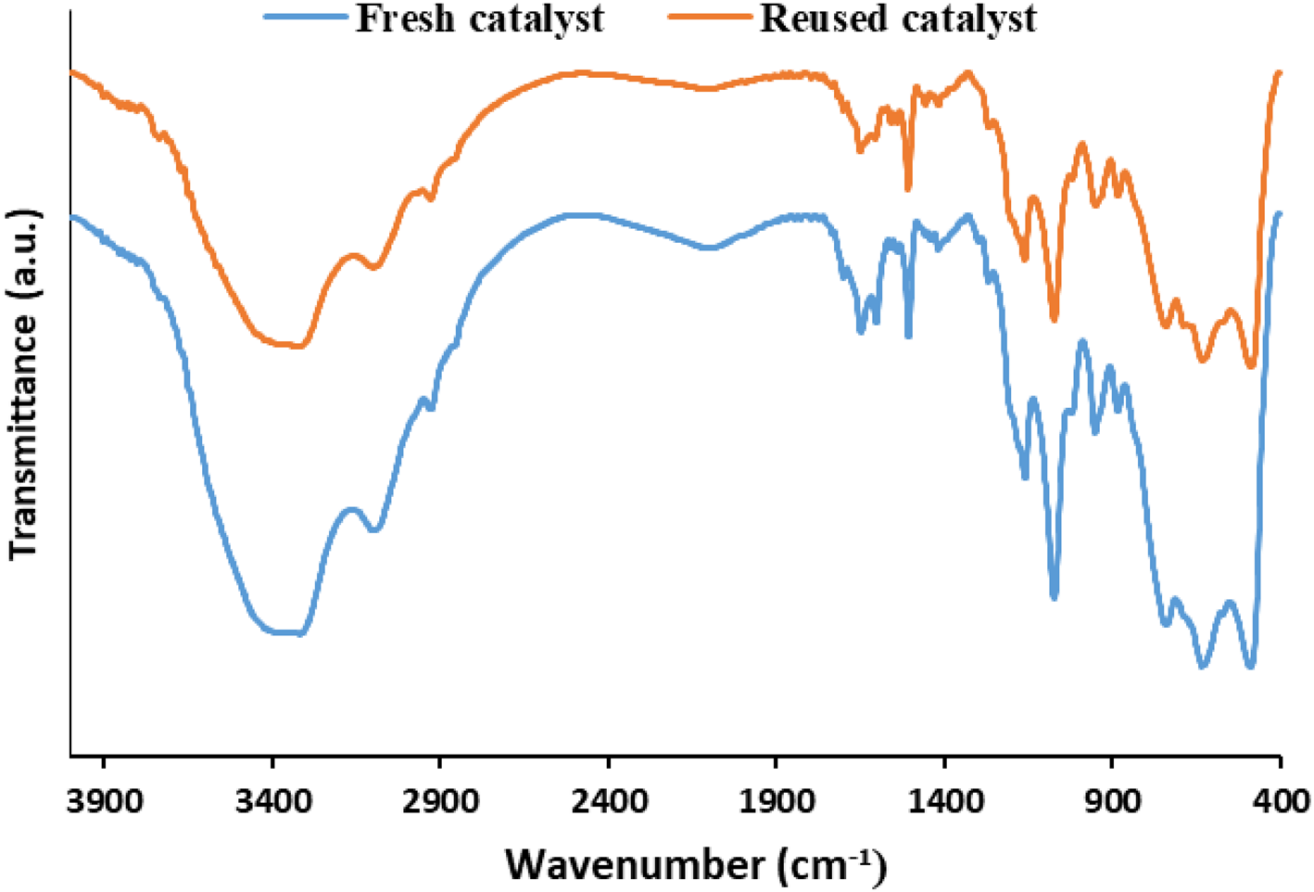
Comparison of the FTIR spectra of fresh and reused Pd/B-CP-HIm after the last run of the model hydrogenation reaction.

The model hydrogenation reaction under the optimized condition was also applied for examining the recyclability of Pd/B-CP-HIm. In this line, Pd/B-CP-HIm was separated from the reaction vessel via centrifugation, washed with EtOH repeatedly and dried at 50 ºC overnight. Afterwards, the recovered Pd/B-CP-HIm was reused for five successive hydrogenation runs. In Fig. 9, the yields of aniline using fresh and recycled Pd/B-CP-HIm are illustrated. According to the results, Pd/B-CP-HIm can be considered as a recyclable catalyst, with only negligible loss of activity upon each run. The reused Pd/B-CP-HIm (the catalyst recovered after the last reaction run) was characterized via FTIR spectroscopy and ICP to assay its stability and Pd leaching respectively. Gratifyingly, the ICP result indicated that Pd leaching that is the origin of loss of the catalytic activity was very scant (~ 1.4 wt.% initial loading of Pd NPs in the fresh Pd/B-CP-HIm). Furthermore, FTIR spectroscopy, Fig. 10, confirmed that both fresh and reused Pd/B-CP-HIm showed similar characteristic absorbance bands in the FTIR spectrum, confirming the chemical stability of Pd/B-CP-HIm upon reusing.

**Figure 11:**
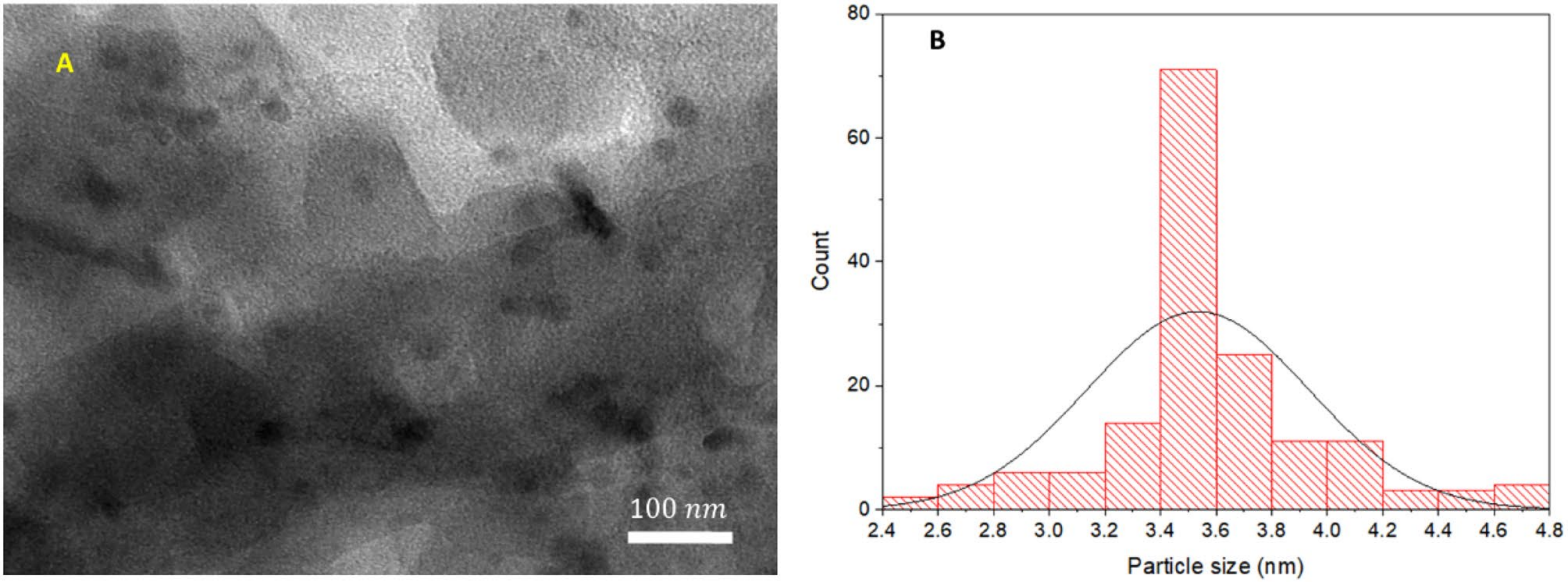
(**A**) TEM image and (**B**) particle size distribution curve for Pd NPs of the recycled catalyst after last run of the reaction.

Next, the effect of recycling on the aggregation of Pd NPs was investigated by recording the TEM images of the recycled catalyst after last run of the reaction. As shown in Fig. 11, upon successive recycling and reuse of the catalyst, very slight aggregation of Pd NPs was observed and the average Pd particle size reached to 3.55 ± 0.4 nm.

### Hot filtration

**Figure 12:**
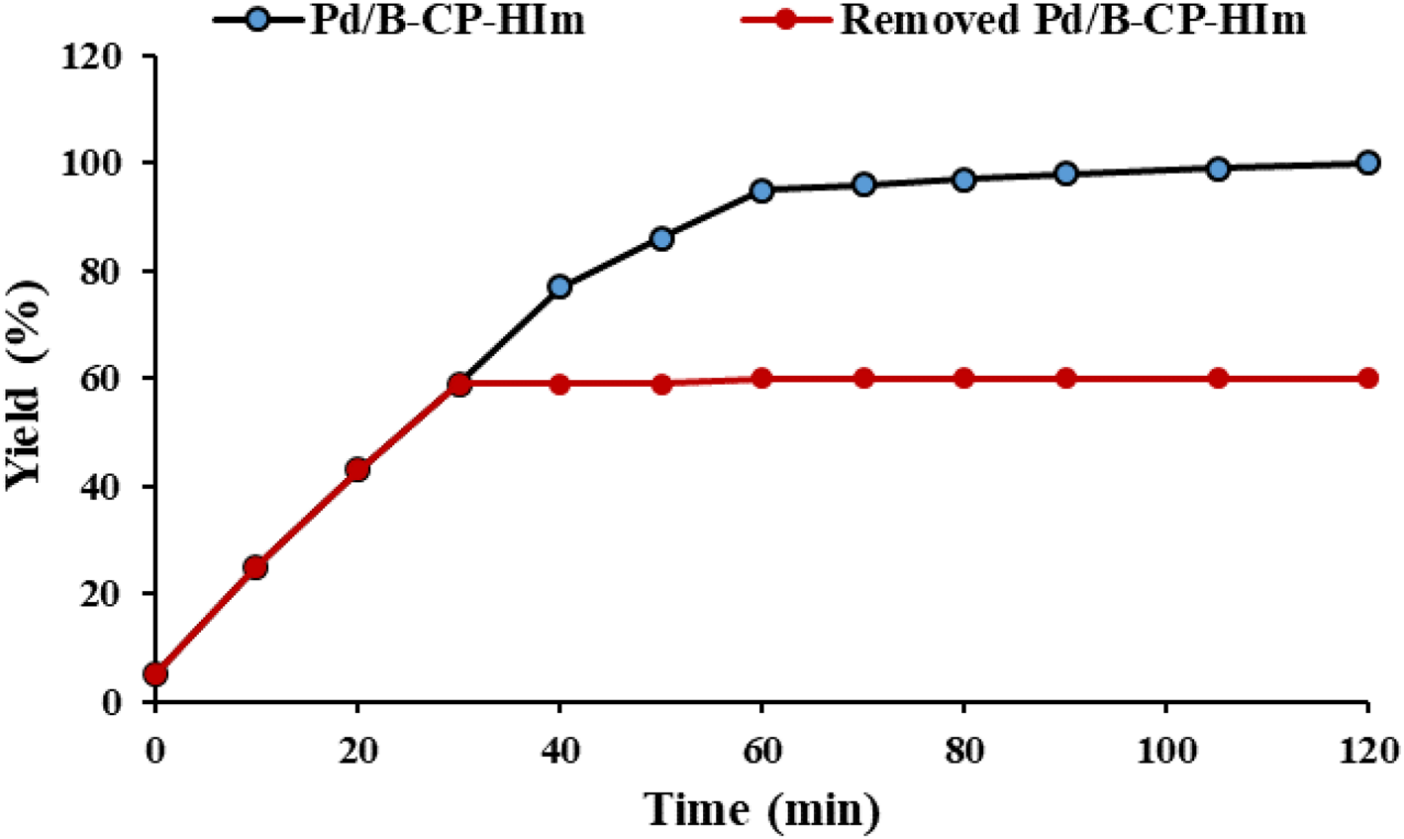
The result of hot filtration test for the model hydrogenation reaction under optimum reaction condition.

One of the important factors in heterogeneous catalysis is stability of the supported catalytic species on the supporting material in the course of the reaction. To examine this issue for Pd/B-CP-HIm catalysis, hot filtration test was applied, in which the catalyst was removed from the reaction vessel after a short time period and the reaction was continued and monitored in the absence of the catalyst to appraise whether any reaction progress was observed in the absence of the catalyst. According to this test, Fig. 12, hydrogenation of nitrobenzene (the model reaction) did not proceed after Pd/B-CP-HIm removal, establishing that Pd NPs were remained stabilized on B-CP-Him in the course of hydrogenation reaction.

### Comparison of the activity of Pd/B-CP-HIm with some other catalysts

Nitro-arene hydrogenation is a fundamental reaction for the synthesis of aniline derivatives. Hence many research groups reported catalytic systems for promoting this reaction under mild reaction condition. As the reaction condition and the nature of the catalyst for each protocol were different, accurate comparison of the catalytic activity of the catalyst is impossible. However, the data in Table 3 indicate that some protocols needed high hydrogen pressure or hazardous solvents that rendered those methodologies less favorable from environmental point of view. On the other hand, for the synthesis of some of the catalytic systems, multi-step synthetic procedures as well as costly raw materials were applied that made the catalyst less economically attractive. Pd/B-CP-HIm, however, is prepared from naturally occurring boehmite and can promote the hydrogenation reaction under mild reaction condition to furnish the desired product in excellent yield. Hence, it can be concluded that Pd/B-CP-HIm can be recognized as a promising catalyst with comparable or superior activity compared to some reported catalysts.

**Table 3:** Comparison of reaction condition and yield of various catalysts for hydrogenation of nitrobenzene.

Entry	Catalyst	Temp. (°C)	Solvent	Time (min)	H2 Pressure	Yield (%)	Reference
1	Pd/B-CP-HIm (0.03 g)	50	H_2_O: EtOH (1:1)	120	1 atm	100	This work
2	Pd/PPh3@FDU‐12 (8.33 × 10^–4^ mmol Pd)	40	EtOH	60	10 bar	99	^30^
3	APSNP^a^ (1 mol%)	r.t	EtOH	120	40 atm	100	^31^
4	Pd@Per-P^b^ (0.03 g)	45	H_2_O: EtOH (1:1)	90	1 atm	98	^32^
5	Pd@CS-CD-MGQDs^c^ (0.5 mol%)	50	H_2_O	60	1 atm	97	^33^
6	PdNP(0.5%)/Al_2_O_3_ (0.3 g)	r.t	THF	180	1 atm	100	^34^
7	Pd@Hal-Hydrogel^d^ + cyclodextrin (2wt%)	50	H_2_O	120	1 bar	95	^35^
8	Pd@Hal-biochar (0.03 mol%)	r.t	H_2_O	60	1 bar	75	^36^
9	Pd@Hal-TCT-Mete	65	H_2_O	75	1 bar	100	^37^

^a^Activated palladium sucrose nanoparticles.

^b^Pd on composite of perlite and polymer.

^c^Pd on composite of magnetic graphitic carbon dot and chitosan-cyclodextrin.

^d^Compositeof halloysite nanoclay and hydrogel.

^e^Halloysite nanoclay decorated with nitrogen containing ligand.

## Experimental section

### Materials and instruments

The developed catalyst in this research, Pd/B-CP-HIm, has been prepared by using the following chemicals and solvents: boehmite, phosphonitrilchloride trimer (PNCT), 4-hydroxybenzaldehyde, (3-aminopropyl) triethoxy silane (APTES), trimethylamine, palladium (Π) acetate, sodium borohydride, tetrahydrofuran (THF), toluene, dimethyl sulfoxide (DMSO), ethyl acetate and methanol (MeOH). Nitro-aromatic compounds and ethanol (EtOH) were also used for performing the hydrogenation reaction. All of the above-mentioned compounds were purchased from Sigma- Aldrich.

The as-synthesized Pd/B-CP-HIm has been analyzed via Fourier transform Infrared spectroscopy (FT-IR, BRUKER TENSOR 35 spectrophotometer 65), X-ray diffraction (XRD, Rigaku Ultima IV instrument with Cu Kα radiation), thermogravimetric analysis under O_2_ atmosphere (TGA, METTLER TOLEDO apparatus), Scanning electron microscopy (SEM, MIRA 3 TESCAN-XMU), elemental mapping and energy-dispersive X-ray analysis (EDS, MIRA 3 TESCAN-XMU), Transmission electron microscopy (TEM Philips EM 208S) and Inductively Coupled Plasma (ICP, Vista-pro instrument).

### Catalyst synthetic procedure

Synthesis of the catalyst was fulfilled via a three-step procedure. First, a ligand, CP-HIm, was prepared and then conjugated on boehmite covalently to furnish a functionalized support for the immobilization of Pd NPs. In the following, each step is discussed in detail.

### Synthesis of CP-HIm

#### Conjugating of 4-hydroxybenzaldehyde to PNCT: synthesis of cyclotriphosphazen-hexa aldehyde (CP-HAL)

CP-HIm was prepared via a two-step procedure. First, 4-hydroxybenzaldehyde was conjugated to PNCT via a known procedure^38^. Briefly, 4-hydroxybenzaldehyde (3.68 g, 30.16 mmol) was dissolved in THF (50 mL) and transferred into a three-necked-flask equipped with a reflux condenser and an Ar inlet. Subsequently, triethylamine (3.05 g, 30.16 mmol) and a solution of PNCT (1.5 g, 4.31 mmol in THF (40 mL)) were added to the flask and Ar gas was purged to the reaction vessel. Then, the reaction temperature was elevated and the mixture was refluxed for 24 h. At the end, the produced tri-ethylamine hydrochloride salt was filtered and the residual solution was concentrated by evaporating the solvent. The main product (CP-HAL) was obtained by adding large amount of distilled water, followed by recrystallization by ethyl acetate^29^.

### Synthesis of cyclotriphosphazen-hexa imine: CP-HIm

Typically, the as-synthesized CP-HAL (1.5 g, 1.74 mmol) was dissolved in THF (30 mL) and then reacted with APTES (2.44 mL, 10.45 mmol) under stirring condition at room temperature for 12 h. The color of the solution was changed and an orange solid was precipitated, which was then filtered, rinsed with EtOH and dried under vacuum.

### Conjugation of CP-HIm on boehmite: synthesis of B-CP-HIm

To covalently graft the as-prepared ligand, CP-HIm, on boehmite, boehmite (2 g) was dispersed in DMSO (20 mL) in a round bottle flask and mixed with a solution of CP-HIm (1.55 g in DMSO (30 mL)) at 100 °C for 24 h. Afterward, the product (B-CP-HIm) was filtered, washed twice with DMSO and THF and dried at ambient temperature overnight.

### Immobilization of Pd NPs on B-CP-HIm: synthesis of Pd/B-CP-HIm

In order to prepare Pd/B-CP-HIm, B-CP-HIm (1.5 g) was dispersed in toluene (20 mL) in a 2-neck round flask under N_2_ atmosphere at ambient temperature and mixed for 30 min. Meanwhile, Pd(OAc)_2_ (0.03 g) was dissolved in toluene (3 mL) and gradually introduced to the B-CP-HIm suspension. After 2 h, a fresh solution of NaBH_4_ (0.2 N in 20 mL MeOH) was dropped to the aforementioned mixture and stirring was continued for 6 h. Finally, the catalyst (Pd/B-CP-HIm) was achieved through filtration and rinsing with toluene, Fig. 1.

### Procedure for the hydrogenation of nitroarenes

To hydrogenize nitro aromatic compounds, Pd/B-CP-HIm (0.03 g) was added to a solution of nitro aromatic compound in 1: 1 mixture of H_2_O/EtOH (7 mL) and hydrogen gas, provided by a hydrogen generator, was purged (P = 1 atm) into the reaction vessel. The reaction temperature was then elevated to 50 °C and the mixture was stirred under the aforesaid condition. Using TLC technique, the progress of hydrogenation reaction was monitored and at the end of the reaction, the mixture was cooled and Pd/B-CP-HIm was separated via centrifugation. To recover Pd/B-CP-HIm, it was rinsed with EtOH three times and dried at 50 ºC overnight. GC analysis was exploited to calculate the yield of the obtained aromatic amine.

## Conclusion

Pd/B-CP-HIm catalyst was fabricated through covalent grafting of the as-prepared CP-HIm ligand on boehmite, followed by palladation via wet impregnation method. The catalyst showed excellent activity and selectivity for hydrogenation of nitroarenes under mild reaction condition in aqueous media. The generality study confirmed that Pd/B-CP-HIm could catalyze hydrogenation of various nitro arenes with electron donating and withdrawing groups. On the other hands, in the case of substrates with –C = O functionality, only reduction of nitro groups was observed, confirming high selectivity of the catalyst. Pd/B-CP-HIm was also recyclable and only negligible Pd leaching and loss of the activity were observed upon recycling. Furthermore, using hot filtration test it was established that Pd/B-CP-HIm catalysis was truly heterogeneous.

## Data Availability

All data used and/or analyzed during the current study are presented in the article. To access the data, all can contact Samahe Sadjadi (s.sadjadi@ippi.ac.ir).
